# Molecular Interactions Between Soil-Borne Oomycetes and Plants: Infection Mechanisms, Host Resistance, and Implications for Sustainable Agriculture

**DOI:** 10.3390/plants15030416

**Published:** 2026-01-29

**Authors:** Usama Amin, Maryam Shabbir, Danjie Long, Zonghua Wang, Meilian Chen

**Affiliations:** 1 College of Plant Protection, Fujian Universities Key Laboratory for Plant-Microbe Interaction, Fujian Agriculture and Forestry University, Fuzhou 350002, China; usamaamin158@gmail.com (U.A.); mariiamshabir69@gmail.com (M.S.); longxy00408@163.com (D.L.); 2 College of Materials and Chemical Engineering, Minjiang University, Fuzhou 350108, China

**Keywords:** oomycetes, *Phytophthora capsici*, *Pythium aphanidermatum*, effector proteins, plant immunity, rhizosphere microbiome, sustainable disease management

## Abstract

Soil-borne oomycetes, such as *Phytophthora* and *Pythium* species, are highly destructive pathogens responsible for severe diseases in crops, ornamentals, and natural ecosystems. These pathogens can persist in soil for many years, making them particularly difficult to control. To establish infection, they deploy a diverse arsenal of effector proteins that manipulate host immune responses, disrupt vital cellular functions, and may influence the rhizosphere microbiome to facilitate successful colonization. *Phytophthora* relies heavily on RxLR effectors to disrupt intracellular immunity, while *Pythium* species predominantly deploy necrosis-inducing NLPs and cell wall-degrading enzymes, with no confirmed canonical RxLR effectors, suggesting distinct evolutionary strategies. This review aims to explore the detailed mechanisms of plant-pathogen interaction. In recent years, significant progress has been made in understanding the molecular dialogue between pathogens and their hosts, particularly how pathogenic species such as *Phytophthora* and *Pythium* manipulate plant immunity through effector secretion, and how plants counteract by activating defense mechanisms at molecular, cellular, and biochemical levels, including changes in hormone signaling, reactive oxygen species (ROS) dynamics, and defense gene expression. The review also outlines emerging disease management strategies and integrative approaches guided by effector biology and microbiome insights.

## 1. Introduction

Soil-borne oomycetes, such as *Phytophthora* and *Pythium*, cause harmful effects to nursery plants, agricultural crops, and woody plants in natural environments [1]. Chytridiomycota fungi and most oomycetes produce biflagellate zoospores that exhibit enhanced motility in water films, enabling effective dispersal and host infection. Consequently, these pathogens are highly effective in warm, moist soils and greenhouse environments, where diseases such as damping-off, root rot and stem blight are most prevalent. Their long-lasting presence in soil and ability to rapidly infect susceptible plants make them difficult to manage, especially under intensive cultivation systems [2]. The *Phytophthora* pathogen is hemi-biotrophic, causing infection and reducing plant defenses before progressing to a destructive necrotrophic stage [3]. However, *Pythium* spp. are necrotrophic and rapidly kill host tissue, leading to damping-off [4,5].

Soil-borne oomycetes, such as *Phytophthora capsici*, significantly reduce production and quality in vegetable crops and affect a variety of horticultural crops. *P. capsici* is particularly known for causing blight in pepper plants, infecting roots, crown, leaves, and fruit, primarily through water splashes [6]. Meanwhile, *Pythium aphanidermatum* is an important cause of seedling damping-off and root rot, especially in greenhouse-grown vegetables [4]. Fungicides have been proven effective in managing and reducing soil-borne diseases caused by oomycetes [7]. However, multiple studies have found fungicide resistance in both oomycetes [8,9]. The increasing risk of fungicide resistance, along with the negative effects of chemical treatments on human health, has created an urgent need for environmentally friendly disease management strategies [10].

To identify environmentally friendly alternatives, we need a better understanding of the biology and pathogenicity mechanisms of soil-borne oomycetes. Effectors (secreted proteins or small molecules that modulate host processes to facilitate colonization) play a crucial role in host-pathogen interactions [11]. Effectors including proteins, RNAs and metabolites, have a specific role in suppressing immunity in leaves and vascular tissues [12]. Recently, attention has shifted toward soil-borne pathogens, where effector activity at the root-soil interface may impact the rhizosphere microbiome [13]. Effector-driven suppression of plant immunity can alter the surrounding microbial ecosystem by actively modifying root exudation profiles, redox status, and cell wall integrity. In soil-borne diseases such as those caused by *Phytophthora* and *Pythium*, where infection occurs in close proximity to root and soil biota, this raises important concerns about how pathogenic effectors influence microbiome assembly and function [11,14].

This review synthesizes current understanding of effector-mediated pathogenicity in soil-borne oomycetes with three priorities. First, we consolidated structural and functional advances on core effector families such as Arginine-any amino acid-Leucine-Arginine (RxLR), crinkler (CRN) and Nep1-like protein (NLP), emphasizing their targets in host immunity and cell biology. Second, we integrated rhizosphere ecology, outlining how effector activity intersects with root exudation, redox/cell-wall dynamics, and microbiome assembly to shape disease outcomes. Finally, we evaluated the translational landscape, considering how effector knowledge can guide sustainable disease management through effector-informed resistance, diagnostics, and microbiome interventions, while aligning with resistance-management principles for existing chemistries. In doing so, we proposed a shift from a pathogen-centric to an ecosystem-centric framework that treats the rhizosphere as a co-determiner for the safe cultivation of agricultural crops. Despite their promise, RNAi-based spray technologies face several limitations, including rapid degradation of dsRNA under field conditions, variable uptake efficiency, potential off-target effects, and challenges in achieving consistent disease control across diverse environments. These constraints highlight the need for improved delivery systems and large-scale field validation before widespread agricultural deployment.

## 2. Classification and Structure of Oomycete Effectors

Pathogens secrete effectors into both the extracellular (the plant apoplast) and intracellular (the plant cytoplasm) environments of plants. Apoplastic effectors occur within the apoplast, where they can block host enzymes or activate extracellular immune responses [15], but they may also remain attached to the pathogen’s cell wall [16]. In contrast, cytoplasmic effectors are delivered into the host cell’s interior, where they can localize to specific subcellular compartments and directly impact host immunity. Effector translocation into host cells is well known in bacterial, nematode, and insect pathogens [17,18,19]. Pathogenicity in oomycetes depends on both apoplastic and cytoplasmic effectors (Table 1). For example, in *P. capsici*, the apoplastic effector PcCBP3 is secreted into the plant extracellular space and recognized by host immune receptors, highlighting the importance of spatial context in effector activity.

### 2.1. Cytoplasmic Effectors Targeting Host Immunity and Vesicle Trafficking

RxLR effectors are cytoplasmic proteins produced by multiple oomycetes, especially many *Phytophthora* species. These effectors contain a conserved RxLR-dEER motif that enables them to enter host cells, likely through phosphatidylinositol-3-phosphate (PI3P)-mediated uptake [21,33]. They are delivered by haustoria and target intracellular defense hubs, such as transcriptional mechanisms and vesicle trafficking pathways, to suppress plant immunity [34]. *Phytophthora* genomes typically contain 300–700 RxLR genes, located in dynamic, repeat-rich regions that facilitate effector evolution [35,36]. Structural research has shown that many of these effectors have a stable WY fold, even in the absence of classical RxLR motifs [37].

The RxLR effector Pc12 from *P. capsici* exhibits a complex virulence mechanism by hijacking host vesicle trafficking. Unlike conventional RxLRs, which suppress immunity via NLR signaling pathways, Pc12 circumvents these defenses by inducing necrosis through an endoplasmic reticulum (ER) stress response. It binds directly to Rab13-2, removing it from the Golgi and increasing its interaction with Rab escort protein 1 (REP1), thereby disrupting secretory protein trafficking and causing ER congestion. Structural studies indicated that Pc12 targets a key Rab13-2 residue; mutation of this residue reduces infection without impairing natural Rab13-2 activity. While RxLRs were previously considered insufficient in *Pythium* species. However, a recent relaxed-motif search had identified 359 RxLR-like candidates in nine genomes, many of which have signal peptides, EER motifs, and disorder-rich domains [34]. Here, “RxLR-like” refers to proteins that contain relaxed or partial RxLR/EER-like motifs and predicted signal peptides, but which lack experimental validation for host translocation and therefore do not meet the criteria of canonical RxLR effectors defined in *Phytophthora*. Although they differ from *Phytophthora*, some *Pythium* RxLRs induce immunological responses in *Nicotiana benthamiana*, indicating that they are functional effectors. These findings expand the evolutionary footprint of RxLRs and confirm their ancestral presence in oomycetes.

### 2.2. Nuclear-Targeting and Transcription-Modulating Effectors

Crinkler (CRN) effectors are an essential type of cytoplasmic effector found in many oomycetes. They were first identified in *P*. *infestans* as host-translocated proteins capable of inducing cell death in plants. They are characterized by conserved N-terminal LFLAK and HVLVxxP motifs that facilitate translocation into host cells, and various C-terminal domains that determine effector function [24,38]. CRNs are modular, and their C-terminal domains often exhibit nuclear localization and DNA-binding characteristics, suggesting roles in regulating host gene expression and enhancing virulence [23].

The initial CRNs, Crn1 and Crn2 were identified using high-throughput functional screening of *P. infestans* secreted proteins. Their expression in plants resulted in distinctive “crinkling and necrosis” phenotypes, giving rise to the CRN name. Subsequent research showed that many CRNs target the host nucleus and disrupt gene expression to increase virulence [39]. Several CRNs in *P. capsici* and *P. infestans* have been experimentally shown to affect host nuclear activities, often resulting in programmed cell death or immune suppression [40]. The gene *PcCRN4* has been identified as a candidate CRN effector in *P. capsici*, produced during infection and anticipated to target the host nucleus, indicating a similar nuclear-targeting mechanism and a possible role in virulence [41]. In *P. cinnamomi*, 25 CRN effectors were identified using HMM-based analysis and classified as either triggers or suppressors of host cell death, demonstrating their dual role in modifying plant immunity [42].

While RxLR effectors are numerous in many *Pythium* species, CRN effectors appear to be more broadly distributed across oomycete lineages based on comparative genomic and motif-prediction analyses, although functional validation remains limited. A recent genome-wide study of *P*. *aphanidermatum* identified CRN-encoding genes with canonical LxLYLAR/K and HVLVxxP motifs, suggesting that *Pythium* species use CRNs as functional analogs of RxLRs for host manipulation [43]. These CRNs are predicted to exist in the secretome, and many exhibit intracellular localization, consistent with typical CRN activity [43]. The absence of canonical RxLR effectors in *Pythium* species, supports the idea that CRNs may represent a more fundamental and widespread effector mechanism across oomycetes.

### 2.3. Necrosis-Inducing and Apoplastic Effectors

Necrosis and ethylene-inducing peptide 1-like proteins (NLPs) are a class of secreted proteins found in bacteria, fungi, and oomycetes. They are best known for their ability to cause host cell death and induce immune responses in dicotyledonous plants [25]. NLPs are categorized into three types based on the number of conserved cysteine pairs in their N-terminal regions: type 1 has one pair, type 2 has two pairs while type 3 has three pairs [25]. Most oomycete plant pathogens, including *P. sojae*, *Pythium ultimum*, and *P. aphanidermatum*, predominantly encode type 1 NLPs, which are typically cytotoxic and serve as pathogen-associated molecular patterns (PAMPs), activating PTI (PAMP-triggered immunity) [5]. Despite their common function in inducing cell death, the involvement of NLPs in virulence appears to be situation-dependent. NLPs are not essential for infection in pathogens such as *Zymoseptoria tritici* and *Botrytis cinerea*, but in *P. capsici* and *Pythium* species, they contribute to disease progression and pathogen proliferation [44]. The genus *Phytophthora* has the largest number of NLP copies, with 33 recognized NLPs and 37 pseudogenes in *P. sojae*. Necrotrophic pathogens, such as *P. ultimum* (six NLPs), exhibit much lower copy numbers [5]. In addition to NLPs, *P. capsici* produces other necrosis-inducing proteins that contribute to its virulence. For example, 12 PcNpp genes are expressed during infection, although their precise roles in virulence remain unknown [45]. Additionally, *PcPME* genes are induced during infection, and exposure of plant tissue to PcPme proteins leads to rapid tissue collapse and cell death [46]. The classification of oomycete effectors highlights their extensive structural and functional diversity: apoplastic effectors trigger necrosis, while cytoplasmic effectors suppress trafficking and immunity. These effectors serve as powerful tools that enable the pathogen to attack the plant host and overcome its immune defense.

## 3. Effector-Mediated Immune Suppression and Host Defense Activation

**Figure 1:**
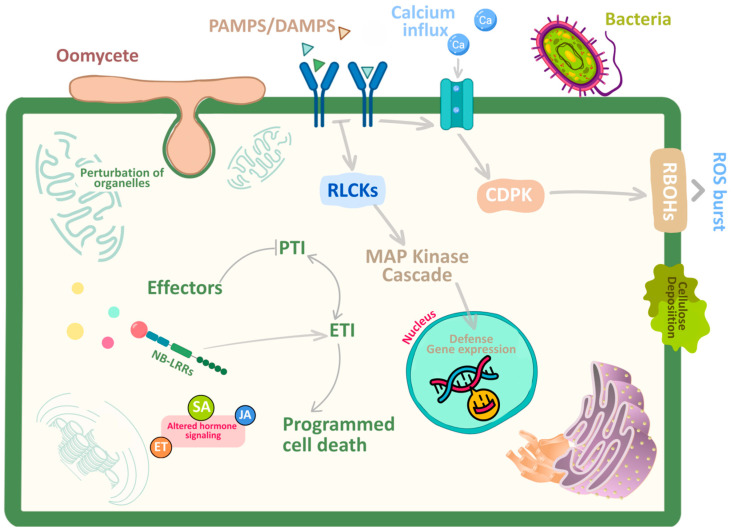
Molecular overview of plant immune signaling and effector interference by pathogens. This figure depicts the two tiered plant immune system pattern triggered immunity (PTI) and effector triggered immunity (ETI) and shows how pathogens influence both responses. PTI begins when cell surface pattern recognition receptors (PRRs) detect pathogen or damage associated molecular patterns (PAMPs/DAMPs), activating downstream signaling components such as receptor like cytoplasmic kinases (RLCKs), calcium dependent protein kinases (CDPKs), and MAP kinase cascades. These events combined cause conventional PTI outputs including as calcium influx, reactive oxygen species (ROS) generation via RBOHs, callose deposition, and transcriptional activation of defence genes. Pathogens such as oomycetes, fungi, and bacteria use effector proteins, which are often translocated through specialised structures like haustoria, to target important immune nodes and modify host cellular processes. Effector actions can alter hormone signaling networks, impair organelle integrity, and reduce early immune signaling, all of which increase susceptibility. When effectors are recognized by intracellular nucleotide binding leucine rich repeat (NB LRR/NLR) receptors, ETI is activated, resulting in enhanced defence responses, widespread transcriptional reprogramming, and, in many circumstances, programmed cell death, which limits pathogen colonization. The graphic depicts the dynamic interplay between immune activation and effector mediated repression across several cellular levels.

Plants and pathogens are engaged in a constant evolutionary arms race. Many plants are naturally resistant to most infections; however, some pathogenic bacteria can cause severe disease. A plant’s primary defense against pathogenic invasion is its innate defense layer, which includes the cell wall and pre-formed metabolites [47] (Figure 1). The first layer of inducible defense is pattern-triggered immunity (PTI), which is activated by PAMPs, such as fungal chitin and bacterial flagellin, as well as damage-associated molecular patterns (DAMPs), including plant peptides and oligosaccharides. These patterns are recognized by plasma membrane pattern recognition receptors (PRRs), which include receptor-like kinases (RLKs) and receptor-like proteins (RLPs) [48]. PRRs activate downstream signaling cascades that trigger rapid immune responses, including calcium influx, mitogen-activated protein kinase (MAPK) activation, reactive oxygen species (ROS) production, and the synthesis of various signaling molecules and phytohormones such as salicylic acid, jasmonic acid, and ethylene [49,50,51,52]. To evade PTI, pathogens have developed effector proteins that interfere with PRR perception or downstream signaling pathways. This interference is called first-level effector-triggered susceptibility (ETS). Effectors can act extracellularly to prevent PRR-PAMP binding or can be transported into plant cells to directly impair PRR signaling components [53,54]. When plants detect intracellular effectors using nucleotide-binding leucine-rich repeat receptors (NLRs), they activate effector-triggered immunity (ETI), the next major immune layer. NLRs detect effectors through direct binding or indirect recognition of altered host targets [55]. ETI often results in a significant transcriptional response and hypersensitive response (HR), a localized cell death that inhibits pathogen transmission [56]. Pathogens can counteract ETI by modifying their effectors, either by altering existing effectors or by deploying new effectors that suppress NLR signaling, a phenomenon known as second-level effector-triggered susceptibility (ETS) [57]. This ongoing molecular conflict between PTI and ETI systems and pathogen effectors drives much of the dynamic interaction between plants and oomycetes, such as *P. capsici* and *P. aphanidermatum*.

### 3.1. Effector-Mediated Immune Suppression

A well-known example of effector-mediated suppression of plant immunity is the RxLR effector PcAvh1 from *P. capsici*. PcAvh1 is highly expressed during early infection and translocates into host cells. Inside the host cell, it binds to the protein phosphatase 2A scaffolding subunit A (PP2Aa), disrupting immune signaling, impairing PTI responses, and promoting pathogen colonization [6]. In addition to PTI suppression, PcAvh1 has also been reported to induce HR-like cell death and to have a dual function in regulating ETI. Transient expression triggers a strong HR-like response in *N. benthamiana*, comparable to a known oomycete elicitor [58]. As in tomato, PcAvh1 shows conserved recognition in the *Solanaceae* family by causing vigorous cell death. In contrast, bell pepper, a natural host of *P. capsici*, exhibits only moderate necrosis during compatible infection with PcAvh1, indicating a balanced interaction [59]. CRISPR-Cas9 mutants show reduced pathogenicity, while overexpression increases disease symptoms, confirming its role in effector-triggered susceptibility [22]. Therefore, PcAvh1 serves as a model for how RxLR effectors target essential host regulators to weaken plant immunity, highlighting a broader effector convergence on conserved signaling hubs.

In contrast to PcAvh1, the Pc12 effector, which manipulates host vesicle trafficking, exemplifies a complex virulence system of *P. capsici.* Pc12 circumvents these protections by inducing necrosis through an ER stress response [60]. Rab GTPases are essential regulators of vesicle trafficking that ensure proper protein transport between the plasma membrane, Golgi apparatus, and ER [61]. Rab13, in particular, facilitates the trafficking of proteins necessary for defensive responses and cell wall integrity [62]. According to a structural study, Pc12 targets an essential residue in Rab13-2; a mutation in this residue reduces infection without impairing the function of native Rab13-2 [63].

Another virulence strategy employed by *P. capsici* is CRISIS2 (CRinkling- and necrosis-Inducing Suppressor 2), an apoplastic RxLR-like effector that targets both PTI and ETI pathways by binding to the plant plasma membrane H^+^-ATPase (PMA). Apoplastic pH homeostasis maintains the balance of acidity in the extracellular space between plant cells [64]. PMAs typically pump protons out of the cell, causing the apoplast to become acidic [65]. This acidification is necessary to activate the pattern-recognition receptor (PRR) signaling, including reactive oxygen species (ROS) production, receptor-ligand binding, and callose deposition [53]. Recent studies have demonstrated the role of the CRISIS2 effector in *N. benthamiana*. The results showed that CRISIS2 binds to PMAs, disrupts proton movement, causes the apoplast to become alkaline, reduces PRR signaling, and ultimately weakens plant immunity [66].

The NLP effector represents another immune suppression mechanism, suppressing plant defense by directly targeting the plasma membrane. Glycosylinositol phosphorylceramides (GIPCs) are abundantly present in dicot plants and are the major sphingolipids in the plasma membrane [67]. GIPCs consist of a ceramide backbone bound to inositol phosphate and one or more sugar residues [68]; they play a pivotal role in maintaining membrane stability, signaling, and charge balance, and act as receptors for NLP toxins [69]. Binding studies and X-ray crystallography have shown that the cytotoxic NLP Pya from the oomycete *P. aphanidermatum* binds to GIPCs in the plant plasma membrane and forms temporary pores that cause ion leakage and cell death. This weakens the plant’s cellular barrier, allowing pathogens to enter and colonize [70]. Similar results for *P. capsici* NLP effectors were observed in *N. benthamiana*. Expression of NLP effectors such as PcNLP2 and PcNLP6 was strongly induced during infection and promoted necrosis. Exogenous dsRNA silencing of PcNLP2 and PcNLP6 significantly reduced disease severity, demonstrating their involvement in immune suppression and pathogenesis [71].

### 3.2. Host Transcriptional Responses and Defense Genes

Plants undergo significant transcriptional reprogramming during infection, which triggers secondary metabolism, defense-related genes, and hormone signaling pathways, including salicylic acid (SA), jasmonic acid (JA) and ethylene (ET). This information is vital for understanding host–pathogen interactions [72]. In the *Solanaceae* family, crops such as potato, tomato, and pepper display conserved defense gene activation patterns during interactions with oomycete pathogens, including upregulation of pathogenesis-related genes, hormone-related defense signaling, and WRKY transcription factors [73]. Among these, pepper plants have been widely studied because they are natural hosts of *P. capsici* and because *Phytophthora* blight causes significant economic losses in pepper worldwide [2]. Several resistance-related genes in pepper (*Capsicum annuum*) have recently been shown to play a role in the response to *P. capsici* infection. Functional analysis revealed that *CaPTI1*, a PTI-related gene, is most highly expressed in roots and is significantly activated not only by *P. capsici* but also by other signaling molecules. Functional silencing of *CaPTI1* by VIGS impaired plant defense; the plants showed reduced root activity and downregulation of critical defense genes such as CaPR1, CaDEF1, and CaSAR82. Consequently, the plants became more susceptible to infection [74].

In addition to transcriptional regulators, resistance gene analogs also contribute to plant defense in response to *P. capsici*. *CARGA2* is a clear example in pepper plants that encodes an NBS-LRR-type protein and shows significant transcriptional activation in response to *P. capsici* infection [75]. A previous study demonstrated that silencing *CARGA2* compromises plant immunity and ultimately increases disease severity. This knowledge is particularly useful in breeding efforts aimed at generating resistant cultivars [76]. Pepper plants also utilize B-box (BBX) zinc finger proteins in defense regulation. Recent research identified *CaBBX14* as closely related to immunological responses; during *P. capsici* infection, *CaBBX14* expression gradually increased, indicating a time-dependent regulatory role in defense pathways [77].

Transcription factors are not only responsible for activating defense but can also negatively regulate plant immunity to balance growth and stress responses [78]. *CaSBP08*, a nuclear-localized SBP-box transcription factor, regulates susceptibility in pepper during *P. capsici* infection. Experimental evidence showed that silencing *CaSBP08* in pepper improves resistance to *P. capsici*, while overexpression of *CaSBP08* in *N. benthamiana* increased susceptibility to *Phytophthora* blight. These data confirm its role as a transcriptional repressor of host defense [79]. Both *CaWRKY01-10* and *CaWRKY08-4* were demonstrated to be positive regulators of plant immunity via transient overexpression analysis. And the stable overexpression of both *CaWRKY01-10* and *CaWRKY08-4* was shown to enhance resistance in transgenic *N. benthamiana*. Moreover, RNA-seq, dual-luciferase reporter, and CUT&RUN-qPCR assays revealed that overexpression of these genes activates several defense-related genes including *PR1*, *PR2*, and other pathogen-responsive genes in *C. annuum*. However, it is necessary to investigate interactions between these proteins to better understand the mechanism behind observation [80]. In soybean, resistance to *Pythium* spp., particularly *P. aphanidermatum*, can be achieved through both vertical and quantitative protection measures. The Rpa1 gene confers R-gene-mediated vertical resistance, while several QTLs contribute to partial immunity or tolerance, demonstrating the genetic complexity of host defense mechanisms against oomycete infections [81]. Understanding this mechanism is essential for disease management. This interaction illustrates how effectors undermine both PTI and ETI, while plants respond by activating defense-related genes that restrict pathogen infection.

## 4. Biochemical Manipulation of Host Immunity by Oomycete Effectors

*P. capsici* and other soil-borne oomycetes secrete a variety of effectors that damage essential components of the host immune response. These effectors target a wide range of molecular pathways, including hormone signaling, ROS generation, vesicle trafficking, and transcriptional control [14]. By targeting these pathways, oomycete effectors reprogram host physiology and suppress immune defenses, allowing the pathogen to establish infection at multiple levels [82].

### 4.1. Targeting Salicylic Acid (SA) Signaling and Immune Regulators

Salicylic acid (SA) is a major phytohormone that regulates both local defense and systemic acquired resistance (SAR) in plants [83]. It enhances resistance to biotrophic and hemibiotrophic infections by activating defense through *NPR1*, a master regulator that translocates to the nucleus and stimulates pathogenesis-related (PR) genes [84]. The RxLR48 effector was identified from *P. capsici* in which it binds to NPR1 to promote pathogen infection. It enhanced NPR1 nuclear localization and inhibited proteasome-mediated degradation to prevent SA signaling by maintaining NPR1 in the nucleus. Additionally, RxLR48 reduces PTI, explaining its role in immune suppression [85]. Genetic evidence showed that another effector, PcAvh103, from *P. capsici*, promoted virulence by targeting the host immunological regulator EDS1 (Enhanced Disease Susceptibility 1). PcAvh103 binds to the lipase domain of EDS1, thereby disrupting its interaction with PAD4 (Phytoalexin Deficient 4), a crucial component of immunological signaling. This disruption reduced both basal and ETI. The EDS1-PAD4 complex functions in conjunction with the SA pathway, helping to enhance SA-related resistance gene expression. PcAvh103 compromised SA-associated defenses, ultimately promoting *P. capsici* infection [86].

Expression analysis revealed that CaBBX14, a B-box transcription factor in pepper, is strongly upregulated during *P. capsici* infection and SA treatments. Functional silencing of CaBBX14 increased susceptibility, lowered SA levels by downregulating PR and SA-related genes, and emphasized its role in SA-mediated defense [87]. Transcriptomic analysis showed that the resistant genotype A204 had early activation of defense responses, including induction of subtilisin-like protease and xylem cysteine proteinase 1, which are implicated in pathogen detection. Calcium- and SA-mediated signaling pathways were significantly activated, resulting in increased expression of defense-related genes, cell wall reinforcement, and flavonoid production. In contrast, the susceptible genotype A198 showed late and reduced stimulation of these responses [88].

### 4.2. Disrupting Membrane Signaling and Vesicle Trafficking for Immune Evasion

Effector-mediated suppression is essential for overcoming host defenses. Plant cells rely on the PMA to maintain proton gradients and membrane potential, which are necessary for early defense signaling, including calcium influx and ROS generation [89]. The RxLR effector CRISIS2 from *P. capsici* targets this enzyme by binding to its C-terminal regulatory domain (specifically in ATPases such as PMA3), thereby inhibiting proton efflux and causing membrane depolarization. This alteration suppresses PTI and facilitates pathogen colonization. For example, CRISIS2 induces host cell death independently of NLR signaling, indicating its dual role in immune suppression and increased virulence [66]. Functional characterization of the *P. capsici* effector RXLR242 revealed its critical role in promoting infection by targeting host RAB GTPases. It inhibits PR1 secretion by binding to RABE1-7 and disrupts the localization of the Flagellin-Sensing 2 (FLS2) receptor by interfering with RABA4-3. This reduces immune signaling and enhances pathogen colonization [89]. Similarly, partial resistance in pepper provides limited selection pressure, allowing severe infection by *P. capsici.* An RNA-seq study showed that the pathogen adapts by upregulating lipid biosynthesis genes and altering the expression of genes involved in nucleic acid metabolism and transport. The RxLR effectors CUST_2407 and CUST_16519 trigger host-specific responses, such as hypersensitive cell death or leaf abscission, which reduce pathogen distribution. These findings suggest that host-induced food limitation is an important defense strategy for generating long-term resistance [90].

However, despite advances in understanding *P. capsici* effector actions, comparative biochemical research on *Pythium* species, particularly pathogenic strains such as *P. aphanidermatum*, remain rare. The biological methods by which these infections alter host immunity are still poorly understood. Unlike *Phytophthora*, *P. aphanidermatum* appears to rely primarily on secreted cell wall-degrading enzymes, such as GH6/GH7 cellulases and pectin lyases, to penetrate host tissues and cause infection. Unexpectedly, no canonical RxLR effectors were found in its genome, suggesting a potentially under-explored mode of host manipulation that differs from known intracellular effector mechanisms [43]. In the case of *Pythium oligandrum*, a non-pathogenic oomycete, it helps plants defend themselves by secreting Nep1-like proteins (PyolNLP5 and PyolNLP7) that function as microbe-associated molecular patterns (MAMPs) [91]. Genomic and functional analyses identified that PyolNLP5 and PyolNLP7 from *P. oligandrum* exhibited strong necrosis-inducing activity and suppressed *P. capsici* infection in solanaceous plants. Protein infiltration and fruit assays demonstrated that these NLPs enhanced immunity and reduced disease severity in tomato and pepper plants by activating defense genes without triggering a reactive oxygen species burst [92]. These strategies suggest that oomycete effectors are not simply agents that suppress the host but can also modulate plant immunity at multiple levels by disrupting the SA pathway, vesicle trafficking, plant hormones, and enzymatic activities. Beyond direct biochemical manipulation of host immune pathways, emerging evidence suggests that effector-mediated immune suppression also influences belowground interactions, particularly within the rhizosphere microbial community.

## 5. Crosstalk Between Immune Suppression and Rhizosphere Microbiota

**Figure 2:**
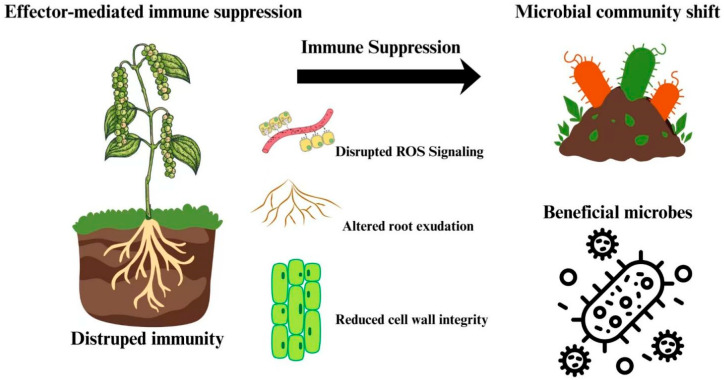
Impact of effector-mediated immune suppression on plant–microbe interactions. This diagram illustrates how pathogen effectors suppress plant immune responses, leading to disrupted reactive oxygen species (ROS) signaling, altered root exudation, and reduced cell wall integrity. These immune changes shift the composition of the microbial community, often favoring the proliferation of beneficial microbes in the rhizosphere.

Soil microorganisms are an extremely diverse and essential component of terrestrial ecosystems, where they maintain soil fertility, promote plant growth, and regulate plant diseases [93]. One of the most fascinating topics in microbial ecology is the composition and function of rhizosphere microbes, which are prominent microbial hotspots [94]. A variety of microbes living in the rhizosphere, protect plants from pathogenic invasions and facilitate their uptake of nutrients from the soil [95]. Plants selectively influence rhizobacterial assemblages in the bulk soil pool to acquire particular functional features necessary for plant health [96]. Consequently, the rhizosphere microbiome significantly increases the plant’s functional repertoire [97]. However, effector-mediated immune systems can indirectly target plant cellular processes in the rhizosphere within the microbial ecosystem. The suppression of PTI and ETI responses compromises root surface integrity and alters the secretion of specialized metabolites, including flavonoids, amino acids, and organic acids that shape microbial recruitment [47]. These changes disrupt the balance between beneficial and harmful microbes, potentially favoring pathogens while suppressing key mutualists, such as plant growth-promoting rhizobacteria (PGPR) and arbuscular mycorrhizal fungi [98]. Immune suppression also interferes with root ROS signaling and the expression of defense-associated genes, further affecting the rhizosphere’s redox status and microbial compatibility [99] (Figure 2). Although clear evidence from oomycetes is lacking, new results from *P. infestans* suggest a strong connection. In potato plants, immune suppression through effector secretion modifies the root exudate profile, reducing the prevalence of beneficial bacteria such as *Pseudomonas* and *Streptomyces*, while increasing taxa that support infection [100]. These changes are associated with weakened SA signaling and altered redox homeostasis, indicating that *P. infestans* manipulates the rhizosphere to promote colonization. This theory was supported by complementary research in *P. cinnamomi*-avocado systems. Infection under disease-suppressive conditions decreased *Proteobacteria* and increased *Actinobacteria* and *Chloroflexi* in the rhizosphere. Changes in beneficial genera such as *Pseudomonas* and *Burkholderia* indicate that *P. cinnamomi* can reshape microbial ecosystems through immune suppression or altered metabolite production [101]. These findings suggest that other oomycetes, such as *P. capsici* and *Pythium* spp., may use similar strategies to create favorable microbial environments. Furthermore, immune suppression can alter the nature of plant-microbe interactions, shifting from mutualistic coevolution to random or competitive colonization. When plant communication fails, microbial community structure becomes less predictable, which may allow commensal bacteria such as *Pythium* spp. or *Ralstonia* to adopt harmful lifestyles [102,103].

This demonstrates the chain reaction in which immune suppression not only aids pathogen survival but also reshapes the microbial community, with lasting consequences for plant health. As our understanding grows that oomycete effectors not only manipulate host immunity directly but also affect the rhizospheric microbial community, further research is needed to clarify the exact mechanisms by which effectors suppress rhizosphere microbes.

## 6. Implications of Disease Management

Oomycete effectors have important implications for plant health, crop productivity, and agroecosystem stability, prompting a re-evaluation of disease management strategies. Integrating this knowledge is essential for improving resistance breeding and biocontrol strategies.

### 6.1. RNAi-Based Spray Technologies for Pathogen-Specific Control

RNA interference (RNAi) is a conserved gene-silencing mechanism in eukaryotes that consists of two fundamental phases. First, Dicer or Dicer-like (DCL) enzymes convert double-stranded RNA (dsRNA) into small interfering RNAs (siRNAs), which are then amplified by RNA-dependent RNA polymerases (RdRPs) to produce secondary siRNAs. Second, these siRNAs are loaded into Argonaute (AGO) proteins, forming the RNA-induced silencing complex (RISC). The guide siRNA strand is used to recognize and cleave complementary target mRNAs [104]. This method provides a sequence-specific approach for regulating gene expression and protecting against infections [105]. Plants may use RNAi to target oomycete diseases such as *Phytophthora*. During infection, host-derived siRNAs are transported to the pathogen via extracellular vesicles and silence critical virulence genes. Disruption of siRNA synthesis in the host increases disease susceptibility, but the pathogen’s effector PSR2 counteracts this protection by inhibiting the plant’s RNAi machinery. These findings support the development of species-specific RNAi-based foliar sprays as a novel, environmentally friendly approach to integrated oomycete disease management [106,107].

*P. capsici* can interfere with siRNA-mediated silencing from the environment. RNA-seq and qRT-PCR studies showed that four RxLR effector genes are highly expressed during infection, with RXLR1 and RXLR4 associated with increased pathogenicity. Virus-induced gene silencing of these effectors in pepper plants significantly reduced disease severity in *Capsicum* and *Nicotiana*. Additionally, co-silencing RXLR1 and RXLR4 enhanced the protective effect [108]. Double-stranded RNA (dsRNA) targeting *P. capsici* effector genes is a potential technique for RNAi-based disease management in solanaceous crops. For instance, the synthesized dsRNAs that specifically target the *P. capsici* effector genes PcNLP2 and PcNLP6. These genes encode necrosis- and ethylene-inducing peptide 1-like proteins, which are critical for pathogenicity. *N. benthamiana* leaves were treated with these dsRNAs, which greatly reduced *P. capsici* infection and lowered expression of both effector genes. Furthermore, suppressing PcNLP2 and PcNLP6 affected host defense gene expression, suggesting increased immunological activation. These findings indicate the effectiveness of dsRNA sprays as a non-transgenic, gene-specific approach for managing oomycete infections in crop systems [71].

Through different defense mechanisms, RNAi-mediated silencing of the potato susceptibility genes StDND1, StDMR1, and StDMR6 improves resistance to *P. infestans*. Regardless of the cause of ROS or cell death, resistance in StDND1-silenced plants is associated with decreased pathogen penetration. In contrast, silencing StDMR1 and StDMR6 activates the SA and ethylene signaling pathways and leads to early ROS accumulation and cell death at infection sites [109]. These results suggest the potential of RNAi sprays as a targeted and long-term strategy for controlling oomycete infections. RxLR effector gene silencing by RNAi protects plants from the oomycete pathogen *P. capsici*. Despite their promise, RNAi-based spray technologies face several limitations, including rapid degradation of dsRNA under field conditions, variable uptake efficiency, potential off-target effects, and challenges in achieving consistent disease control across diverse environments. These constraints highlight the need for improved delivery systems and large-scale field validation before widespread agricultural deployment.

### 6.2. Rethinking Resistance Breeding with Effector and Microbiome Data

Resistance breeding in *C. annuum* against *P. capsici* presents significant challenges due to the polygenic and race-specific nature of host resistance. Although several resistance loci and R genes have been identified, particularly from donor lines such as CM334, their integration into elite cultivars often faces obstacles such as epistatic breakdown, linkage drag, and phenotype-genotype mismatches, limiting the effectiveness of marker-assisted selection [110,111]. *P. sojae* resistance breeding in soybeans presents the importance of coordinated approach. The combination of Rps genes (e.g., Rps1a, Rps6) with QTLs for partial resistance has greatly increased resistance durability and yield under infection pressure [112,113]. CRISPR/Cas9 genome editing technologies have enabled the production of chimeric NLR clusters, such as Rps1, thereby broadening the resistance spectrum and providing a blueprint for comparable applications in pepper [114].

This cross-species defense highlights the importance of identifying *N. benthamiana* accessions carrying the immune receptor NbPrf, which specifically recognizes the conserved RxLR effector AvrNb from *P. sojae*. If the plant loses this receptor, it becomes susceptible to *P. sojae*, *P. capsici*, and *P. infestans* [115]. This cross-species defense confirms the role of common effectors in identifying targets for resistance breeding. Similar advancements in *Pythium* resistance breeding, including the discovery of QTLs in soybean and maize involved in the RPSR1 locus and its associated gene *ZmPEPR2*, demonstrate the importance of PTI and multi-pathogen defense pathways [116,117]. Overall, these findings call for a paradigm shift in resistance breeding, one that combines effector biology, sugar-responsive signaling, NLR gene innovation, and microbiome-aware selection to create broad-spectrum and long-lasting resistance pipelines against oomycete infections.

### 6.3. Biocontrol Agents Resilient to Effector-Mediated Shifts

Pathogen-secreted effectors not only damage host immune components but also affect the rhizosphere environment, mostly reducing the viability of traditional biocontrol techniques. However, numerous microbial strains have shown resistance to these effector-mediated problems, and they have emerged as promising components of integrated disease management. *Trichoderma* spp., particularly *T. harzianum*, *T. viride*, and *T. longibrachiatum*, have demonstrated prolonged antimicrobial activity against *P. capsici* and *Pythium* spp., even when host immunity is compromised [118,119]. These strains act through various mechanisms, including the secretion of antifungal metabolites (such as trichomycin and pentaibols), the production of cell wall-degrading enzymes, and the regulation of induced systemic resistance (ISR) pathways involving JA, SA, and ethylene signaling [120,121,122].

Similar resistance has been provided by other microbial groups. *Bacillus amyloliquefaciens* treatment of soybeans reduced *P. sojae* infection by increasing ROS generation and callose deposition [123]. *Paenibacillus polymyxa* SC09-21 significantly decreased disease severity by increasing the activities of defense enzymes such as phenylalanine ammonia lyase (PAL), peroxidase (PO), polyphenol oxidase (PPO), and superoxide dismutase (SOD) in pepper [124]. Furthermore, co-inoculation of *Bacillus vallismortis* TU-Orga21 with *B. subtilis* TU-Orga1 resulted in a 350% increase in iturin, a production and complete suppression of *P. aphanidermatum*-induced damping-off in kale, achieving results comparable to chemical control with metalaxyl [125]. These examples demonstrate that synergistic microbial consortia can provide effective biocontrol even in effector-disrupted soil conditions. Resilient biocontrol materials that function in effector-altered microbiomes to initiate plant defenses have become essential for long-term disease management. Integrating such bacteria into microbiome-informed IPM provides an environmentally friendly defense against *P. capsici* and other oomycetes.

### 6.4. Role of Plant Growth Regulators (PGRs)

PGPR enhance plant growth and development through various processes, including increased mineral nutrient availability and phytohormone regulation [126]. Endophytic *Cronobacter* spp. JZ38 improves salt stress tolerance and development in *Arabidopsis thaliana* through direct contact and volatile emissions. In vitro studies have shown that these volatiles reduced *P. infestans* growth by up to 58% without adverse effects [127]. Similarly, screening of chili rhizosphere revealed that *Pseudomonas* and *Bacillus* spp. are highly antagonistic to *P. capsici*. These PGPR strains reduced *P. capsici* infections by 52–63% while simultaneously promoting chili plant development [128]. These bacteria produced high levels of hydrogen cyanide (HCN), catalase, indole-3-acetic acid (IAA), and siderophores [129]. Notably, Imperata cylindrica roots function as PGPRs, significantly reducing the severity of *P. infestans* infection in potatoes. This effect is quite distinct from that of bamboo-root PGPR. The observed disease reduction has been correlated with the production of antibiotic secondary metabolites [130]. In general, effector-informed resistance breeding, strong biocontrol agents, and microbial metabolite-based strategies, when used with essential agronomic techniques such as crop rotation, reduced tillage, soil solarization, and precision fungicide application, contributing to achieve sustainable disease management. While chemical inputs such as metalaxyl and captan are still necessary during periods of high disease pressure, their careful and resistance-aware application is essential for maintaining efficacy and reducing environmental impact [131,132]. Conservation tillage and rotational diversity have also demonstrated potential in altering *Phytophthora* life cycles and influencing beneficial rhizosphere microbes [133]. Similarly, soil solarization and biofumigant amendments show a promising synergistic effect with microbial inoculants by reducing damping-off incidence and improving soil health.

**Figure 3:**
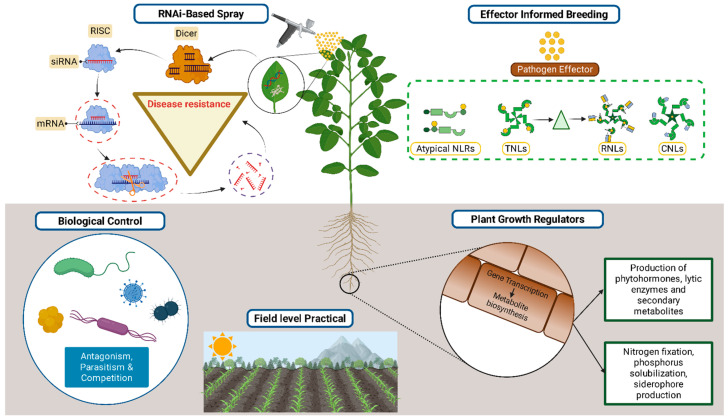
Implication of Disease management. Integrated approaches for managing plant diseases, including RNAi-based spray technologies, resistance breeding, biocontrol agents, and cultural practices. Beyond antagonism, microbial chemistry is also highlighted as an emerging tool for enhancing plant protection. Together, these strategies illustrate a holistic framework aimed at reducing pathogen pressure, improving crop resilience, and promoting sustainable agricultural systems.

All these components work together to develop a microbiome-informed and evolutionarily resilient framework for next-generation integrated pest management (IPM) (Figure 3).

## 7. Conclusions and Future Perspectives

Soil-borne oomycetes such as *P. capsici* and *P. aphanidermatum* are serious pathogens that exploit plant immunity and the surrounding microbiome to establish effective infections. These pathogens deploy a diverse arsenal of effectors, including RxLRs, CRNs and NLPs, to suppress host defenses, alter hormone signaling, and manipulate root exudates, ultimately modifying the rhizosphere to favor their survival. The involvement of these effectors in host manipulation and microbial disruption has broadened the scope of plant-pathogen interactions from a primarily immunological perspective to a wider ecological context. Conventional disease management practices need to be reevaluated based on this new understanding. To effectively utilize insights from effector biology, transcriptome responses, and resistance breeding, it is necessary to proceed after selecting important R genes. The ability of beneficial microbes to produce bioactive compounds that improve soil health and plant defense, as well as their resistance to effector-mediated immune suppression, is also changing biocontrol strategies. Functional studies on *Pythium* effectors, particularly CRNs and NLPs, have been extremely limited, and their roles in virulence and host specificity remain unclear. Furthermore, while effectors have been found to control root exudation and immunological responses, their immediate effects on beneficial microorganisms, such as mycorrhizal fungi and plant growth-promoting rhizobacteria, are not well understood. The efficient molecular mechanisms by which soil-borne oomycetes impact microbial community assembly remain elusive. Advanced techniques such as gnotobiotic systems and synthetic microbial communities (SynComs) provide promising platforms for resolving the complex three-way interactions among plant, pathogen, and microbiota. However, these methods have received little application in bacterial or fungal systems and have not yet been applied to oomycetes. Similarly, multi-omics approaches, such as metagenomics, transcriptomics, proteomics, and metabolomics, are infrequently used in oomycete pathosystems, particularly during root colonization stages, even though effector activity is critical.

In order to move towards next-generation IPM, these gaps need be filled up by embracing effector-informed resistance breeding, using biocontrol agents that survive immunological disruption, and utilizing microbial metabolites with established anti-oomycete activity. To develop sustainable and evolutionarily resilient management techniques, these biological innovations must be integrated with fundamental agronomic approaches such as crop rotation, conservation tillage, soil solarization, and resistance-aware fungicide applications. Therefore, understanding *Phytophthora* and *Pythium* and inhibiting their ecological influence using multi-disciplinary and integrated approaches is critical to regulating their impact and maintaining crop health in the era of sustainable agriculture.

Molecular plant pathology, microbial ecology, and systems biology must all be integrated in order to advance our knowledge of soil-borne oomycetes. The thorough functional annotation of effector repertoires across *Phytophthora* and *Pythium* species, including the identification of lineage-specific effectors that might be responsible for host specialisation and differential microbiome manipulation, should be a high priority for future research. Effector-target screening, protein interaction mapping, and dual RNA-seq during root colonization are examples of high-throughput techniques that can offer previously unheard-of insights into the dynamic signaling interactions between plant, pathogen, and surrounding microbial populations. Simultaneously, the use of carefully crafted synthetic microbial communities (SynComs) and regulated gnotobiotic platforms can shed light on how certain microbial taxa or metabolic modules react to perturbations caused by effectors. These mechanistic research findings should be used to construct effector-informed breeding pipelines that take advantage of natural R-gene diversity or engineer broad-spectrum resistance via receptor decoys. In addition, combining rhizosphere metabolomics with ecological modelling will be critical for anticipating how pathogen-induced alterations in root exudation influence community assembly and disease consequences. By combining these multidisciplinary approaches, we will be able to create microbiome-resilient cropping systems and develop long-term, ecologically sound solutions to limit the impact of soil-borne oomycetes.

Future research should focus on identifying novel effector targets in the plant immune system and microbiome, especially those involved in recognition, signaling, and metabolic regulation. Advanced gene-editing techniques, notably as CRISPR/Cas systems, provide new opportunities to examine effector activities using targeted knockouts and allele-swapping assays, allowing for accurate characterization of virulence determinants. Creating high-resolution interaction maps between effector proteins and host cellular components will aid in the development of next-generation resistant cultivars with greater recognition breadth and less sensitivity to effector-driven immune suppression.

Another interesting option is the creation of microbiome-based therapies informed by effector ecology. Beneficial microbial consortia can be rationally created to occupy ecological niches sought by oomycetes, produce antagonistic compounds, or boost plant immunity via priming. Integrating SynCom-guided microbial design with multi-omics monitoring may enable the development of predictive models for disease onset and precision biocontrol deployment. Such frameworks will be required to design disease management strategies that are environmentally sustainable, evolutionarily robust, and field scalable in a variety of cropping systems.

## Figures and Tables

**Table 1:** Comprehensive catalogue of oomycete effector families: structural features, functions, and host targets.

Effector Family	Representative Species	Key Structural Motifs/Features	Secretion/Delivery Pathway	Primary Host Targets	Biological Function(s)	Representative Studies/References
RxLR (cytoplasmic effectors)	*Phytophthora infestans*, *P. sojae*, *P. capsici*	N-terminal signal peptide; RxLR–dEER region; conserved WY/LWY folds; intrinsic disorder in N-termini	Secreted from haustoria; translocated into host cytoplasm (PI3P-linked uptake proposed)	PRR/NLR signaling hubs (e.g., PP2A), MAPK/Ca^2+^ nodes, vesicle trafficking (Rab GTPases), transcriptional regulators	Suppress PTI/ETI, dampen ROS/MAPK outputs, rewire trafficking; some trigger or suppress cell death depending on context	[20,21,22]
CRN/Crinklers (cytoplasmic, often nuclear-targeting)	*P. infestans*, *P. capsici*, *P. cinnamomi*	N-terminal LFLAK and HVLVxxP motifs; modular C-termini with DNA-/chromatin-associated domains; frequent NLS	Secreted from haustoria; host cell import; many accumulate in nucleus	Transcriptional machinery, DNA replication/repair components; PCD regulators	Reprogram host gene expression; induce or suppress PCD; promote colonization during hemibiotrophy	[23,24]
NLPs (Nep1-like proteins; apoplastic cytolysins)	*P. capsici*, *P. sojae*; *Pythium aphanidermatum*	Conserved NPP1/NLP domain; Type I–III (cysteine pair classes); catalytic/metal-binding residues	Secreted to apoplast; remain extracellular	GIPC sphingolipids in eudicot plasma membrane	Pore formation (ion leakage and necrosis): also act as PAMPs to trigger PTI in some hosts	[25,26,27]
Elicitins (sterol-binding proteins; apoplastic)	*P. infestans* (INF1), *P. nicotianae* (ParA1), *P. cinnamomi*	~95–100 aa elicitin domain; disulfide-stabilized; high-affinity sterol binding	Secreted to apoplast; perceived by specific RLPs in Solanaceae	Sterol sequestration; PRR complexes at cell surface	Elicit HR/defense in some hosts; modulate sterol homeostasis and membrane properties	[28,29]
Apoplastic protease inhibitors (EPICs/EPIs)	*P. infestans* (EPIC1/2B; EPI1/10)	Cystatin-like (EPICs) and Kazal-like (EPIs) inhibitor domains	Secreted to apoplast; bind host proteases	Papain-like cysteine proteases (C14/PIP1) and subtilases (P69B)	Inhibit immune proteases that generate/relay defense signals; protect pathogen from apoplastic immunity	[30,31]
Glucanase inhibitor proteins (GIPs)	*P. sojae*, *P. infestans*	Secreted inhibitor proteins; stabilize in oxidizing apoplast	Apoplastic; interact with host β-1,3-glucanases	Endo-β-1,3-glucanases	Block release of β-glucan elicitors; shield oomycete cell-wall β-glucans from hydrolysis	[32]
Small cysteine-rich apoplastic effectors (SCRs)	*P. cactorum* (PcF), *P. infestans* (various SCRs)	Small, secreted, cysteine-rich, disulfide-stabilized; often lineage-specific	Secreted to apoplast; some bind cell walls	Cell wall enzymes/receptors; membrane interfaces	Induce necrosis or suppress apoplastic defenses; contribute to host specificity	[28,29]
Cell-wall-associated adhesion/elicitor proteins (e.g., CBEL-like)	*P. nicotianae* (CBEL), *P. infestans* (CBM-containing adhesins)	CBM1 (cellulose-binding) + PAN/lectin repeats; glycosylated; wall-anchored	Pathogen cell wall/apoplast; surface-exposed	Plant cell wall & PRRs sensing wall perturbation	Adhesion to cellulosic substrates; trigger defense; modulate wall integrity at the interface	[28]

## Data Availability

No new data were created or analyzed in this study. Data sharing is not applicable to this article.
